# None sharp corner localized surface plasmons resonance based ultrathin metasurface single layer quarter wave plate

**DOI:** 10.1038/s41598-021-88540-w

**Published:** 2021-04-26

**Authors:** Qinyu Qian, Pengfei Liu, Li Fan, Liang Zhao, Chinhua Wang

**Affiliations:** 1 College of Physical Science and Technology, Yangzhou University, Yangzhou, 225009 Jiangsu China; 2 School of Optoelectronic Science and Engineering and Collaborative Innovation Center of Suzhou Nano Science and Technology, Soochow University, Suzhou, 215006 China; 3 Key Lab of Advanced Optical Manufacturing Technologies of Jiangsu Province and Key Lab of Modern Optical Technologies of Education Ministry of China, Soochow University, Suzhou, 215006 China

**Keywords:** Nanoscale devices, Optical materials and structures

## Abstract

We report on a non-sharp-corner quarter wave plate (NCQW) within the single layer of only 8 nm thickness structured by the Ag hollow elliptical ring array, where the strong localized surface plasmons (LSP) resonances are excited. By manipulating the parameters of the hollow elliptical ring, the transmitted amplitude and phase of the two orthogonal components are well controlled. The phase difference of π/2 and amplitude ratio of 1 is realized simultaneously at the wavelength of 834 nm with the transmission of 0.46. The proposed NCQW also works well in an ultrawide wavelength band of 110 nm, which suggests an efficient way of exciting LSP resonances and designing wave plates, and provides a great potential for advanced nanophotonic devices and integrated photonic systems.

## Introduction

Wave plates are important optical devices that are used to realize phase modulation and polarization conversion, which is realized by adding different phase delays to the two polarization components perpendicular to each other. Traditional wave plates are mostly manufactured by bulky birefringent crystals, which suffer from depth of phase modulation and difficulties in integration. Recently, as the very fast development of metasurface structures, localized surface plasmons (LSP) resonance based wave plates have been widely researched and published^1–19^. Some other metasurface wave plates such as dielectric metasurface wave plates are also studied^20,21^. The rapid development of nanostructures provides a new way of designing metasurface devices^22–28^. Zhao et al. proposed plasmonic metasurfaces formed by orthogonal elongated nanorods and complementary nanoslits^3^. The structure produces strong LSP resonances at the sharp corners of the rectangular holes. The intensity of the resonances is adjusted by the size of the two rectangular holes to control the phase delays of the two polarization components within the 40 nm-thick silver layer. Li et al. demonstrate a metal/insulator/metal tri-layer structure with L-shaped hole arrays inside to realize the polarization conversion in optical transmissions at near-infrared wavelength in totally 460 nm thicknesses^6^. Strong LSP resonances are excited inside the L-shaped holes around the sharp corners, which contribute to the phase delay of the two polarized components. The x-polarized incident light can be conversed to the y-polarized light by the efficiency of 93% at the wavelength of 1400 nm. Chen et al. proposed a strategy to realize plasmonic quarter-wave plate with subwavelength rectangle annular aperture arrays in the Ag film of 200 nm thickness^7^. The incident light can excite strong LSP resonances around the sharp corners of the rectangles and is located in the different regions in the hole according to the polarization. The transmission of their proposed quarter wave plate is 46% at the wavelength of 1550 nm. A plasmonic quarter-wave plate based on 60 nm thick U-shaped Ag nanopatches array in the near-infrared range is designed and presented by Chen et al.^15^. Strong LSP resonances are excited around different sharp corners of the U-shaped silver nanopatches according to the polarization of the incident light, leading to the different phase delays between x- and y-polarized components. The transmission of 46.3% and phase deference of 1.57 is realized at the wavelength of 1550 nm. Li et al. also proposed a reflective half-wave plate composed of single L-shaped antennas, and a reflective quarter-wave plate composed of double L-shaped antennas^17^. By exciting strong LSP resonances around the sharp corners of the L-shaped antennas, the phases of different polarized light can be delayed differently within totally 600 nm thicknesses. The LSP reonances based quarter-wave plates under oblique incidence are also researched by Ma et al. They develop a plasmonic approach toward broadband infrared polarimetric crypsis based on all-metallic rectangular column array, and realize polarizing camouflage technique^19^. In these LSP based wave plates, the strong LSP resonances are all excited around the sharp corners, leading to the different phase delays of the two mutually perpendicular polarization components. However, the designed structures with sharp corners cannot be manufactured perfectly, but will be prepared as rounded corners, which can extremely weaken the LSP resonances such that the efficiencies of these wave plates will drop significantly as seen in the Part 1 of Supplementary information.

In fact, the LSP resonances can be excited at structures with no sharp corners, such that the performance is less sensitive to the structural errors. In this paper, we proposed a non-sharp-corner quarter wave plate (NCQW) within the single Ag layer of only 8 nm thickness, where the strong LSP resonances are excited in the hollow elliptical ring array. The phase difference between the x- and y-polarized components is from 1.5 to 1.59 and the amplitude ratio is from 0.78 to 1.18 at the wavelength band from 770 to 880 nm. The phase difference of π/2 and amplitude ratio of 1 is realized simultaneously at the wavelength of 834 nm with the transmission of 0.46. This proposed NCQW suggests a new way of exciting LSP resonances and designing wave plates, which has huge application prospects in advanced nanophotonic devices and integrated photonic systems^3,15,18–20^.

### Theory and structure design

**Figure 1:**
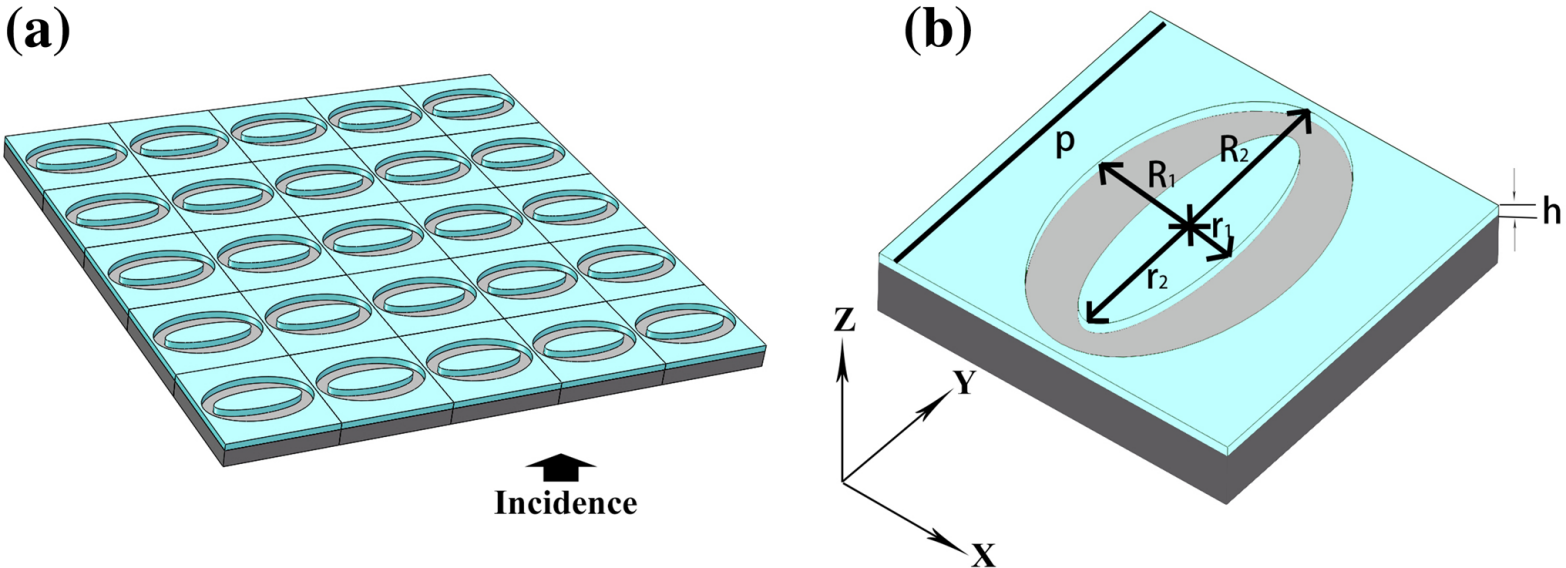
Structure of proposed NCQW. (**a**) Schematics of the proposed plasmonic metasurface NCQW structure (5 × 5 units). The light incidend from the substrate side as the arrow. (**b**) One unit of the proposed NCQW structure.

The structure of the proposed NCQW is shown in Fig. 1. The Ag layer with the thickness of *h* is coated on a SiO_2_ substrate and is shaped to the hollow elliptical ring array. As shown in Fig. 1b, the short and long radius of the inner and outer ellipses are *r*_1_, *r*_2_, *R*_1_, and *R*_2_, respectively, and the period is *p*. A linearly polarized plane wave with prescribed polarization orientation *θ* (as seen in Fig. 1b) with respect to *X* axis is normally incident from the substrate side as seen the arrow in Fig. 1a. The transmission performance and the electric field inside the structures of the proposed NCQW is calculated and optimized using the finite difference time domain (FDTD, Lumerical FDTD Solutions, Canada) method. In the simulation, only one unit cell is in the simulated region and the periodic boundary conditions were set at *x* and *y* boundaries such that the periodic structure can be simulated. It was set as perfectly matched layers condition at z boundaries. A mesh size of 5 nm along all directions is used in the 3D simulation mode. When drawing the distribution of electric fields, the mesh size is set to 1 nm. The accuracy is set to 3, and the auto shutoff min (convergence) is set to 1e-5. In the simulation, light is incident on the structure from the substrate along the Z direction (shown in Fig. 1 a); i.e., the incident angle is 0°.

**Figure 2:**
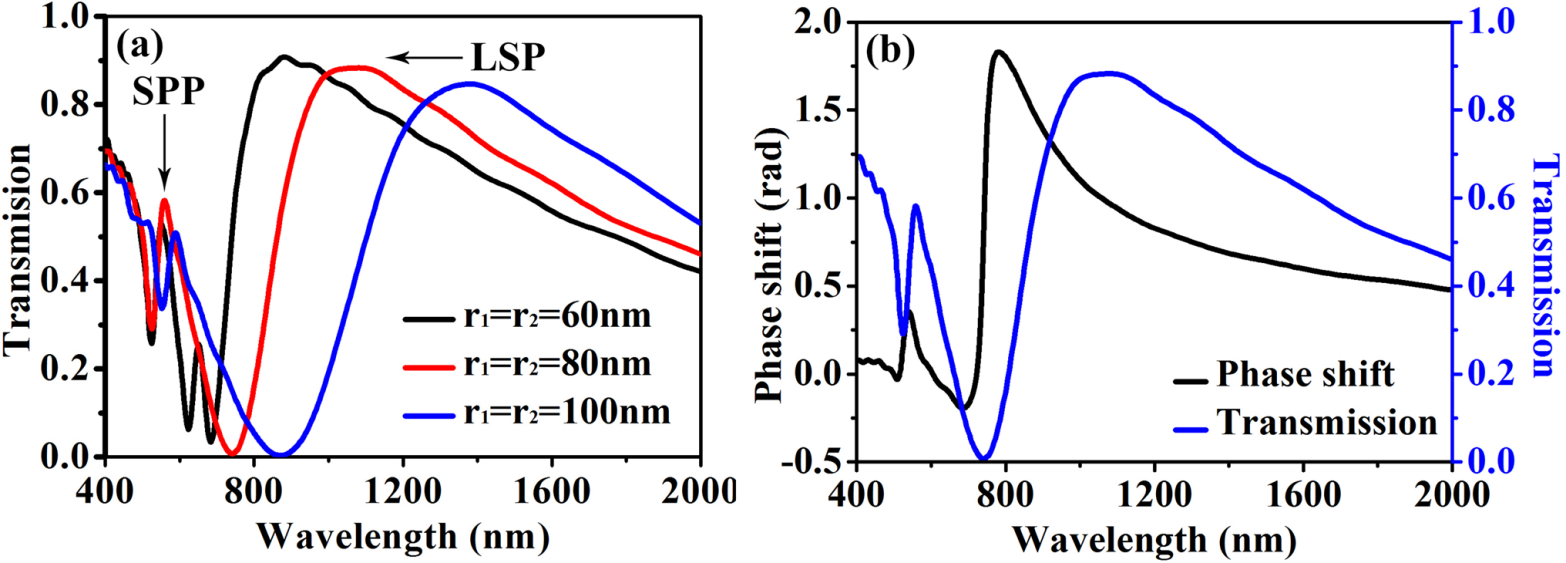
Performance of the hollow ring. (**a**) The transmission at *r*_1_ = *r*_2_ = 60 nm, 80 nm and 100 nm, respectively. (**b**) the transmission and the phase shift at *r*_1_ = *r*_2_ = 80 nm. The other parameters are *R*_1_ = *R*_2_ = 120 nm, *h* = 8 nm, and *p* = 330 nm. Lumerical FDTD Solutions; version: 8.23.2305. www.lumerical.com.

Different from the previous LSP resonances based wave-plate; there are no corners in the proposed structures. In fact, however, the LSP resonances can also be excited inside the non-sharp-corner structures. Figure 2a shows the transmission spectrum ranging from 400 to 2000 nm and with the orientation *θ* = 45° (i.e., *xy*-polarized incidence). In this simulation, we set *r*_1_ = *r*_2_ = 60 nm, 80 nm and 100 nm, respectively, and the other parameters are *R*_1_ = *R*_2_ = 120 nm, *h* = 8 nm, and *p* = 330 nm. It is shown that there is a serious of dips in each transmission spectrum as a result of Wood’s anomaly, and the peaks in each transmission spectrum correspond to excitation of SPP resonances^7^. The LSP resonant peaks aroused by the non-propagating modes supported by the hollow ring can also be seen in the spectrums. The LSP resonant wavelength red-shifts significantly with *r*_1_ (or *r*_2_) increasing (i.e. the width of the hollow ring decreasing), which is a distinctive feature of the LSP resonances. Figure 2b shows the transmission and the phase shift relative to the structures without the Ag layer (i.e., only the substrate) when *r*_1_ and *r*_2_ are fixed at 80 nm. It can be seen that the phase shift reaches approximately π/2 at the wavelength range around 755 nm and 858 nm near the peak of the transmission spectrum, which means that the phase shift of the transmission field can be well controlled by appropriately designing the nanostructures and manipulating the LSP resonances.

**Figure 3:**
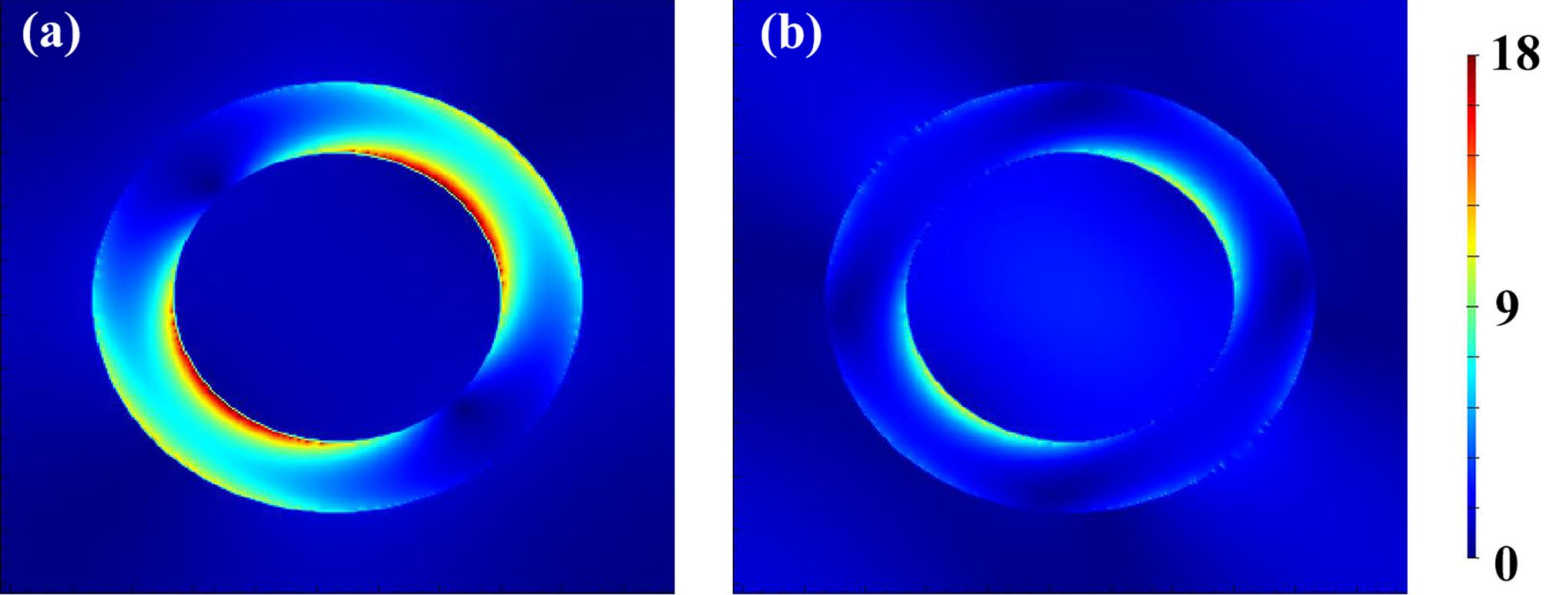
Electric field distribution of *xy*-polarized incident light of the structures in Fig. 2. The wavelength is (**a**) 1075 nm and (**b**) 800 nm, respectively. The parameters are *r*_1_ = *r*_2_ = 80 nm, *R*_1_ = *R*_2_ = 120 nm, *h* = 8 nm, and *p* = 330 nm.

Figure 3 shows the electric fields within the nanopatterns under normal incidence with the orientation *θ* = 45° when *r*_1_ = *r*_2_ = 80 nm (i.e., the red line in Fig. 2a). The wavelength of the incident light is 1075 nm in Fig. 3a corresponding to the peak of transmission in Fig. 2a, and is 800 nm in Fig. 3b corresponding to the deviated wavelength. It is clearly seen that there is resonant field at both the inner and the outer edges inside the hollow ring along the direction of incident orientation, which is much stronger in Fig. 3a than in Fig. 3b. The electric fields better displays the physical mechanism of the LSP resonances induced extraordinary transmission, which occurs only if a matching condition between the metasurface structures and the incident light is satisfied. In other words, the resonant wavelength can be manipulated by carefully regulating the parameters of the nanostructures.

**Figure 4:**
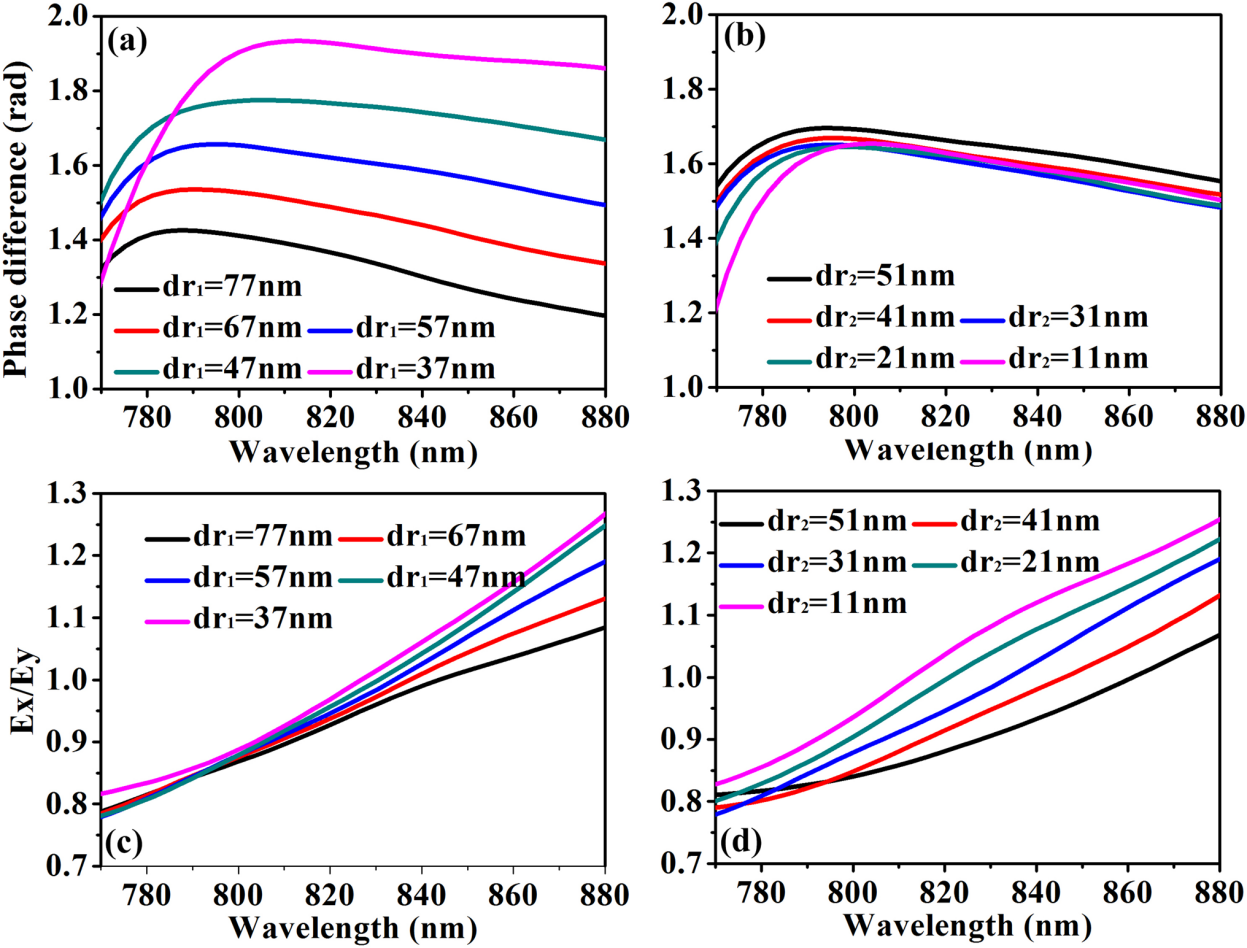
The amplitude ratio and the phase difference. (**a**) The phase difference and (**b**) the amplitude ratio of transmitted light under *xy*-polarized incidence. (**a**,**c**) *dr*_1_ decreases from 77 to 37 nm and *dr*_2_ = 31 nm. (**b**,**d**) *dr*_2_ decreases from 51 to 11 nm and *dr*_1_ = 57 nm. The other parameters are *h* = 8 nm and *p* = 330 nm.

An ideal quarter wave plate requires an amplitude ratio of 1 and a phase difference of π/2, between the transmitted electric fields along *X* and *Y* axes, which require the difference along the two directions in the structures. In the nano-structures with sharp corners, the structures can be divided into horizontal and vertical parts, which can be separately adjusted to precisely control the features of the *x*- and *y*- components of incident light, respectively. However, to simplifying the design and reducing the processing difficulty, the non-sharp-corner nano-structure is seen as a whole such that its physical mechanism is very different from that of the sharp-corner nano-structures. Figure 4 shows the amplitude ratio (*E*_x_/*E*_y_) of the two orthogonal components of electric field along *X* and *Y* axes and their phase difference under different parameters at the wavelength range from 770 to 880 nm with orientations *θ* = 45°, where *dr*_1_ = *R*_1_ − r_1_, and *dr*_2_ = *R*_2_ − *r*_2_. The height *h* of the Ag structures is 8 nm (The effect of different Ag thickness can be seen in the Part 2 of Supplementary information) and the period *p* is 330 nm. Figure 4a shows the phase difference when *dr*_2_ is fixed at 31 nm and *dr*_1_ varies from 37 to 77 nm. For comparison, the amplitude ratio when *dr*_1_ is fixed at 57 nm and *dr*_2_ varies from 11 to 51 nm is shown in Fig. 4b. It is obviously seen that the phase difference at the wavelength of 834 nm rise from 1.30 to 1.87 within *dr*_1_ decreasing, but varies just between 1.59 and 1.61 when *dr*_2_ decreasing. It means that the phase difference is much more sensitive to the change of the structures in *X* direction than that in *Y* direction. It can be also seen that the phase difference does not change much at the wavelength band of 110 nm (For example, the phase difference varies between 1.5 and 1.59 at *dr*_1_ = 57 nm as the blue line in Fig. 4a). The amplitude ratio of the structures in Fig. 4a,b are also investigated as seen in Fig. 4c,d. In contrast to the phase difference, the amplitude ratio is more sensitive to the change of *dr*_2_ than that of *dr*_1_. It is shown in Fig. 4c that the rises from 0.97 to 1.03 at the wavelength of 834 nm within *dr*_1_ decreasing. When *dr*_1_ is fixed and *dr*_2_ decreases as seen in Fig. 4d, the amplitude ratio rises from 0.91 to 1.10 at the wavelength of 834. These characteristics can be advantages in designing the wave plate that the phase difference can be precisely controlled by adjusting the parameters in *X* direction without affecting the amplitude ratio, and as is the parameters in *Y* direction for adjusting the amplitude ratio. In this way, the parameters are optimized at *dr*_1_ = 57 nm and *dr*_2_ = 31 nm to realize the phase difference of π/2 and the amplitude ratio of 1 at the wavelength of 834 nm.

**Figure 5:**
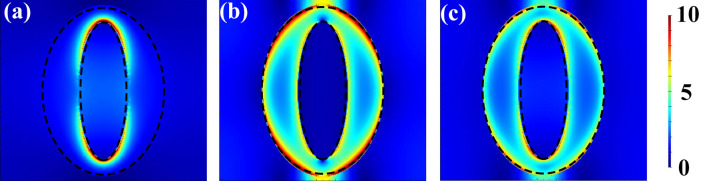
Electric field distribution of the proposed NCQW. (**a**) *x*-polarized, (**b**) *y*-polarized and (**c**) *xy*-polarized incident light at the wavelength of 834 nm. The parameters are *r*_1_ = 40 nm, *r*_2_ = 125 nm, *R*_1_ = 97 nm, *R*_2_ = 156 nm, *h* = 8 nm, and *p* = 330 nm, respectively. Lumerical FDTD Solutions; version: 8.23.2305. www.lumerical.com.

Figure 5 shows the distribution of the electric field inside the structure under the incidence of *x*-, *y*- and *xy*-polarization, where the parameters are optimized at *r*_1_ = 40 nm, *r*_2_ = 125 nm, *R*_1_ = 97 nm, *R*_2_ = 156 nm, *h* = 8 nm, and *p* = 330 nm, respectively. It can be seen that the electric field bounded inside the hollow elliptical ring is stronger in the top and down sides (i.e., along *Y* direction) than in the left and right sides (i.e., along *X* direction) under both *x*- (Fig. 5a) and *y*- (Fig. 5b) incidence. These results explain why the amplitude ratio is more sensitive to the change of parameters along *Y* direction (such as *dr*_2_) than that along *X* direction (such as *dr*_1_). It is shown in Fig. 3 that the strength of the electric field characterizes the strength of LSP resonance, which determines the peak in the transmission spectrum. In contrast, when the parameters along *X* direction (such as *dr*_1_) changes, the LSP resonance is not affected much, such that the amplitude ratio changes very little. On the other hand, the phase difference is more relevant to the effective refractive index, which is influenced by the proportion of Ag and air. The proportion varies much more with the change *dr*_1_ of than that of *dr*_2_, such that the phase difference is much more sensitive to the change of the structures in *X* direction than that in *Y* direction.

**Figure 6:**
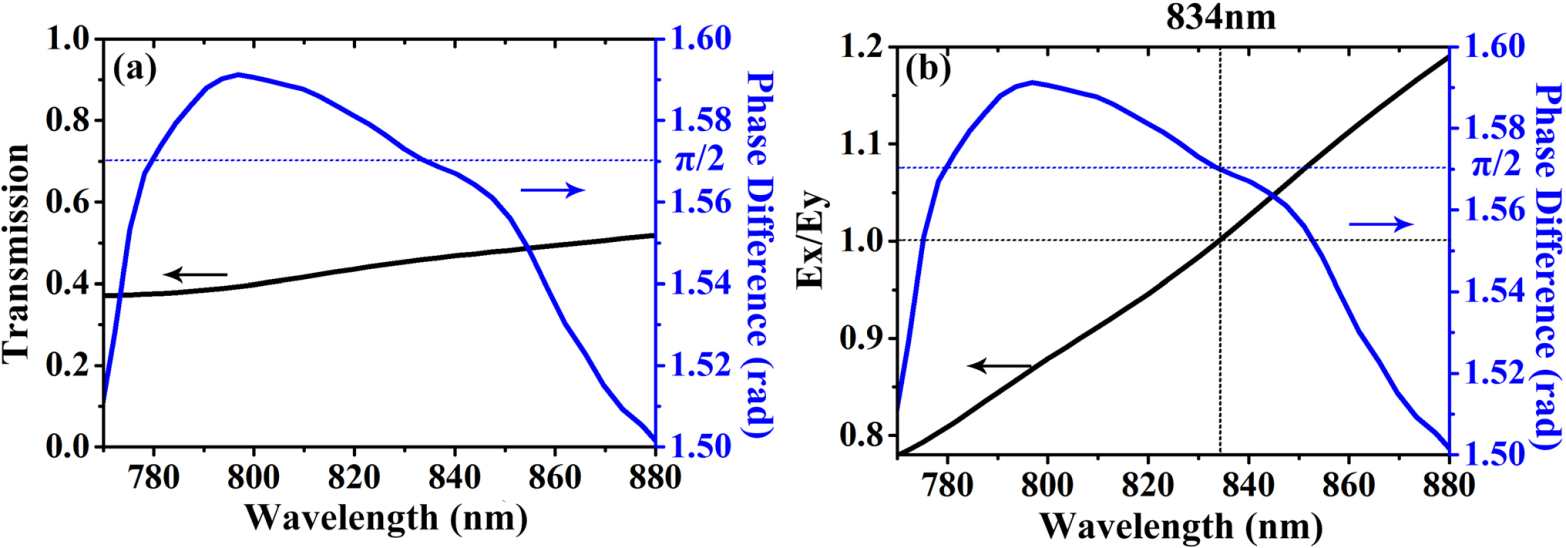
Performance of the proposed NCQW. (**a**) The transmission and phase difference between the transmitted electric fields along *X* and *Y* axes of the proposed NCQW. (**b**) The amplitude ratio of the two orthogonal components of electric field along *X* and *Y* axes and their phase difference of the proposed NCQW.

Figure 6 shows the performance under *xy*-polarized incidence of our proposed NCQW with the optimized parameters same as in Fig. 5. The parameters are optimized based on the results in Figs. 4 and 5. *R*_1_ and *r*_1_ are utilized to control the phase difference, which should be π/2 in the working wavelengths. The amplitude ratio is adjusted by optimizing *R*_1_ and *r*_1_, such that an amplitude ratio of 1 can be realized in the working wavelengths. Figure 6a shows the transmission and phase difference between the transmitted electric fields along *X* and *Y* axes at the wavelength band range from 770 to 870 nm. It is seen that the transmission is from 0.372 to 0.521 and the phase difference is from 1.5 to 1.59. At the wavelength of 834 nm the transmission reaches 0.46 and the phase difference is π/2. It is also seen that a variation of less than 4.5% in phase difference occurs within a very broad wavelength range 770 nm and 870 nm. Figure 6b shows the amplitude ratio of the two orthogonal components of electric field along *X* and *Y* axes and their phase difference, where the amplitude ratio is from 0.78 to 1.18. An ideal quarter wave plate requires an amplitude ratio of 1 and a phase difference of π/2, which can be simultaneously realized at the wavelength of 834 nm as the dashed line seen in Fig. 6b) These results mean that our proposed NCQW works perfectly at the wavelength of 834 nm and works well at the wavelength band range from 770 to 880 nm.

## Discussion

In conclusion, a non-sharp-corner quarter wave plate within the single Ag layer of only 8 nm thickness is theoretically demonstrated, in which the strong LSP resonances are excited in the hollow elliptical ring array. The transmitted amplitude and phase of the two orthogonal components can be precisely controlled by manipulating the parameters of the hollow elliptical ring. The phase difference of π/2 and amplitude ratio of 1 is realized simultaneously at the wavelength of 834 nm with the transmission of 0.46. The phase difference between the *x*- and *y*-polarized components is from 1.5 to 1.59, the amplitude ratio is from 0.78 to 1.18 at the wavelength band from 770 to 880 nm, and the transmission is from 0.372 to 0.521. Different from traditional LSP resonance based quarter-wave plates, there is no sharp corners in our proposed ultrathin structures, such that the efficiency reduction can be avoided when fabricating the structures. It is also noted that the transmission may be increased by further study. Our proposed NCQW can also be easily fabricated by utilizing sputter coating depositions and followed focused Ion beam etching. The proposed NCQW is easy integration with simple structure, which suggests a novel way in exciting LSP resonances and designing wave plates, and has great potentials in advanced nanophotonic devices and integrated photonic systems.

## Supplementary Information

Supplementary Information
