# Supplementary material for: None sharp corner localized surface plasmons resonance based ultrathin metasurface single layer quarter wave plate
Source: Sci Rep. 2021 Apr 26;11:8956. doi: 10.1038/s41598-021-88540-w (PMC8076246; doi:10.1038/s41598-021-88540-w)
Supplement: Supplementary file 1 — Supplementary Information [file 41598_2021_88540_MOESM1_ESM.docx]

**Supporting Information**

**None Sharp Corner Localized Surface Plasmons Resonance Based Ultrathin Metasurface Single Layer Quarter Wave Plate**

**Qinyu Qian, Pengfei Liu, Li Fan, Liang Zhao & Chinhua Wang**

**Part 1**

The LSP based wave plates can excite strong LSP resonances around the sharp corners, leading to the different phase delays of the two mutually perpendicular polarization components. However, the designed structures with sharp corners cannot be manufactured perfectly, but will be prepared as rounded corners, which can extremely weaken the LSP resonances such that the efficiencies of these wave plates will drop significantly.

To better show the performance degradation when the sharp corners are prepared as rounded corners, we simulate the performance of the structures proposed by Chen. et al. with rounded corners. Fig. S1 is the structures proposed by Chen. et al.^1^ The parameters are *P* = 550 nm, *L*_1_ = *L*_2_ = 380 nm, *W*_1_ = 80 nm, *W*_2_ = 46 nm, and the height is 205 nm. It can be seen from Fig. S1(b) and (c) that the corresponding phases of the transmitted field of the two orthogonal components along x and y axes and the phase difference between the two components. It is seen that a phase difference of π/2 can be achieved at the designed wavelength 1.55 μm. The transmission is 0.46 at the wavelength of 1.55 μm. It is shown that their proposed plasmonic quarter-wave plate performs well with sharp corners. However, when the sharp corners are changed to rounded corners as shown in Fig. S2, both the phase difference and the transmission decreases obviously. It is noted that all the parameters are the same as those in Fig S1. It can be seen from Fig. S2(a) that the phase difference is much smaller than that in Fig. S1(b), and reaches the maximum value of 1.35 (much smaller than π/2) at the wavelength of 2220 nm. Fig. S2(b) also shows that the transmission is only 0.388 at the wavelength of 2220 nm. In the LSP based waveplates, the phase difference is caused by the strong resonances around the sharp corners. When the sharp corners are changed to rounded corners, the resonances are inevitably weakened, leading to the lowering of the phase difference.


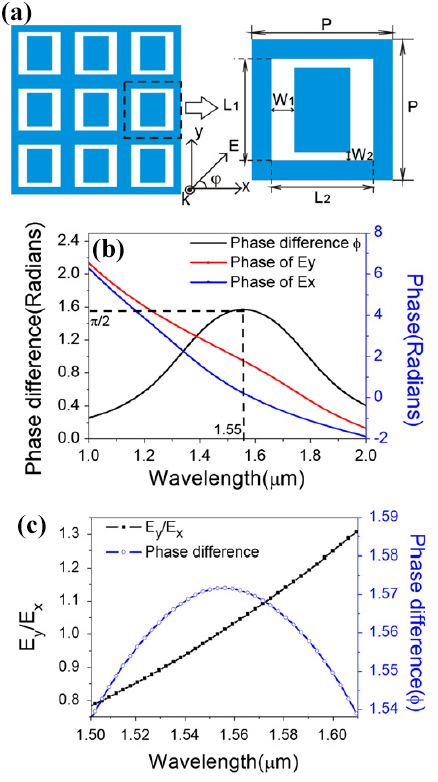


Fig. S1 (a) Schematic of a quarter-waveplate with a patterned Ag film on a SiO_2_ substrate.^1^ (b) Phase spectrums of transmitted electrical components along 0˚ and 90˚ with a linearly polarized incident light.^1^ (c) The detailed amplitude ratio and the phase difference of two orthogonal components of electric field along x and y axes.^1^


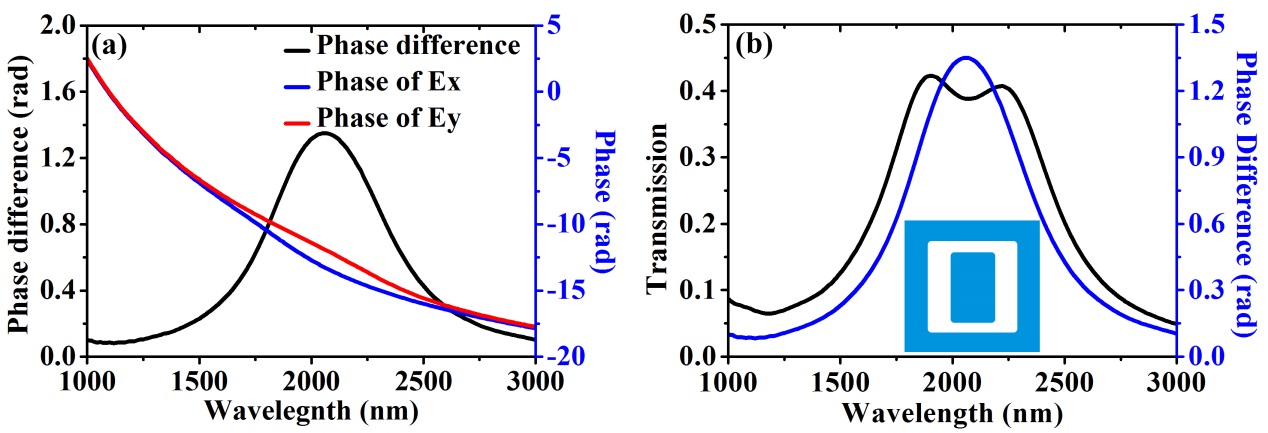


Fig. S2 The performance of the structures in Ref. 1 with rounded corners. (a) Phase spectrums of transmitted electrical components along 0˚ and 90˚ with a linearly polarized incident light. (b) The phase difference and transmission spectrum. The inset in (b) is the schematic of the rounded corner structure. All the parameters are the same as those in Fig. S1.

**Part 2**

Fig. S3 shows the effect of different Ag thickness in the proposed NCQW. It can be seen from Fig. S3(a) that the amplitude ratio is almost unaffected by the height *h*. It can also be seen from Fig. S3(b) that phase difference spectrum blue shifts very little within *h* increasing. In fact, the height hardly affects the performance of the proposed NCQW. Although the height can be as thin as possible in simulations, however, a too thin film will make fabrication much more difficult. Therefore, we set *h* = 8 nm, which can be easily realized in coating process.


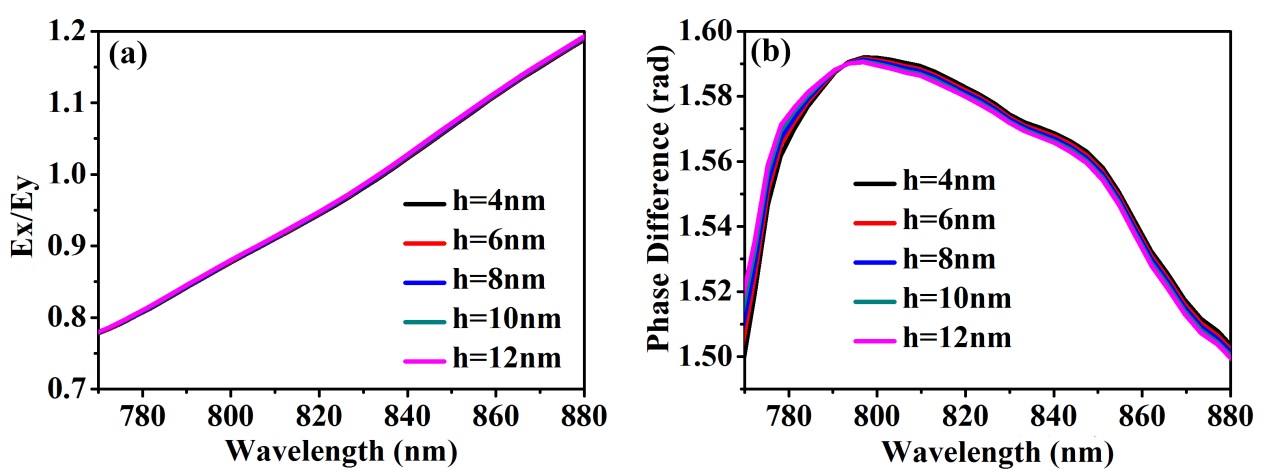


Fig. S3 (a) The amplitude ratio and (b) the phase difference spectrums with different heights.

**References**

1. Chen, Z. H., Wang, C. H., Lou, Y. M., Cao, B., & Li, X. F., Quarter waveplate with subwavelength rectangular annular arrays. Opt. Commun. 297, 198-203 (2013).
